# EGFR-TKI联合放疗治疗晚期非小细胞肺癌的研究进展

**DOI:** 10.3779/j.issn.1009-3419.2014.04.12

**Published:** 2014-04-20

**Authors:** 夏南 李, 广迎 朱

**Affiliations:** 1 100044 北京，北京大学人民医院 Peking University Renmin Hospital, Beijing 100044, China; 2 100142 北京，北京市肿瘤防治研究所，恶性肿瘤发病机制及转化研究教育部重点实验室，北京大学肿瘤医院 Key Laboratory of Carcinogenesis and Translational Research (Ministry of Education), Peking University Cancer Hospital, Beijing 100142, China

**Keywords:** EGFR-TKI, 放疗, 肺肿瘤, EGFR-TKI, Radiotherapy, Lung neoplasms

## Abstract

肺癌是全球最常见的恶性肿瘤之一，严重威胁人类生命。近年来，以表皮生长因子受体酪氨酸激酶抑制剂（epidermal growth factor receptor tyrosine kinase inhibitors, EGFR-TKIs）为首的靶向药物在肺癌治疗中取得巨大进展。因其具有高选择性和低毒性的优势，目前已成为Ⅳ期非小细胞肺癌（non-small cell lung cancer, NSCLC）*EGFR*突变患者的一线治疗方案。然而随着临床的广泛应用，继发耐药成为临床亟待解决的问题。近年来，基础研究证实，EGFR-TKI具有放射增敏性，理论上二者联合不但可以解决放疗后期肿瘤的放射抵抗以及EGFR-TKI继发耐药，还可以增加对肿瘤杀伤能力，同时副反应较同步放化疗小。因此，EGFR-TKI与放疗联合成为晚期NSCLC（Ⅲb期/Ⅳ期）极具探索的治疗模式。本文就EGFR-TKI与放疗联合治疗晚期NSCLC的基础与临床研究进展进行综述。

## Abstract (en)

Lung cancer is one of the most common malignant tumor in the world, severely threatening human life. Recently, targeted therapy such as the epidermal growth factor receptor tyrosine kinase inhibitors (EGFR-TKIs) made huge progress in treatment of lung cancer. EGFR-TKIs, with its high selectivity and low toxicity, is the first choice for *EGFR*-mutated patients in stage Ⅳ non-small cell lung cancer (NSCLC). However, secondary drug resistance becomes a clinical problem to be urgently resolved. In recent years, a series of preclinical studies showed that EGFR-TKI can enhance the antitumor activity of ionizing radiation. Therefore, EGFR-TKI combined with radiation is extremely promising therapy pattern for advanced NSCLC. This review will discuss the research status in EGFR-TKI and radiotherapy for advanced NSCLC.

肺癌是全球最常见的恶性肿瘤之一，其中非小细胞肺癌（non-small cell lung cancer, NSCLC）约占85%。近年来，靶向治疗在NSCLC中取得巨大进展。其中，表皮生长因子受体酪氨酸激酶抑制剂（epidermal growth factor receptor tyrosine kinase inhibitors, EGFR-TKIs）具有高选择性和低毒性的优势，目前已成为Ⅳ期NSCLC中*EGFR*突变患者的一线治疗方案。EGFR-TKI包括吉非替尼、厄洛替尼，通过完全阻断EGFR-TKI细胞内连接三磷酸腺苷的区域，阻止EGFR效应器的自体磷酸化，抑制其激活，从而对癌细胞的增殖、生长、存活等多条信号传导通路起到阻断作用。近年来，基础研究证实，EGFR-TKI具有放射增敏性，理论上二者联合不但可以解决放疗后期肿瘤的放射抵抗以及EGFR-TKI继发耐药，还可以增加对肿瘤杀伤能力，同时副反应较同步放化疗小。因此，EGFR-TKI与放疗联合成为晚期NSCLC极具前景的治疗模式。本文就EGFR-TKI与放疗联合治疗Ⅲb期/Ⅳ期NSCLC的基础与临床研究进展进行综述。

## 1 基础研究

### 1.1 在NSCLC细胞系中EGFR-TKI放射增敏的机制

#### 1.1.1 引起细胞周期阻滞

细胞周期时相分布影响细胞对放射线的敏感性：G_2_/M期最敏感，G_1_期居中，S期最抗拒。基础研究结果显示，吉非替尼可以引起细胞周期的重新分布，使10%-15%的细胞从放射抗拒的S期分布于G_0_/G_1_期，从而使整个细胞群体增加放射敏感性^[1]^。Chinnaiyan等^[2]^使用厄洛替尼及6 Gy放射线处理人NSCLC细胞系（H226），流式细胞仪结果显示，二者单独作用可以分别使细胞停滞于G_1_期和G_2_/M期，并下调处于S期细胞的比例；联合应用时，厄洛替尼可以进一步降低分布于S期细胞的数量。其机制可能在于EGFR-TKI提高了G_1_期CDKs的重要抑制因子p27的表达^[3]^。

#### 1.1.2 诱导细胞凋亡

EGFR-TKI抗肿瘤的作用机制之一，即为促进肿瘤细胞凋亡。Ⅰ期药效动力学试验显示，吉非替尼可以上调p27的表达，从而抑制Cyclin D1并增加凋亡指数^[4]^。多项临床前研究证实，促进凋亡亦为EGFR-TKI提高放射敏感性的重要途径。Tanaka等^[5]^选取了A549和H1299两种NSCLC细胞系进行体外研究。经过浓度仅为1 μmol/L的吉非替尼处理和2 Gy放射线照射后，A549细胞系存活率从50.4%±0.7%降至39.4%±0.8%（*P*=0.05）；H1299细胞系存活率从55.8%±2.5%降至40.9%±0.06%（*P*=0.01）。Chinnaiyan等^[2]^对厄洛替尼与辐射联合作用的基础研究中也同样发现，二者联合可以协同促进H226细胞系的凋亡、明显抑制NSCLC细胞系异体移植于裸鼠的瘤体生长。EGFR-TKI与辐射联合有协同作用，机制考虑可能与其引起肿瘤细胞凋亡减轻瘤负荷从而降低放射线杀灭肿瘤的剂量^[3]^、影响Bcl-2家族^[6]^及上调p27kip1表达增加放射敏感性^[7]^有关。

#### 1.1.3 降低放射抗拒

辐射可以使肿瘤细胞产生TGF-α、激活EGFR-TK继而启动EGFR增殖旁路，并能够增加细胞内EGFR表达，从而产生辐射抵抗。EGFR-TKI可以抑制放射线诱导的EGFR自体磷酸化、阻断EGFR下游信号通路，减低辐射抵抗。Chinnaiyan等^[2]^用2 Gy及10 Gy的射线照射H226细胞系，蛋白印迹分析显示细胞系EGFR自体磷酸化增加。而用1 μmol/L厄洛替尼处理24 h后，这种辐射诱导的EGFR自体磷酸化明显被抑制。Tanaka等^[5]^发现，放射线可以促进A549细胞系EGFR下游信号分子pEGFR、pAKT的表达，而吉非替尼可以抑制这种作用。另外，放射线能够提高A549、H1299细胞系pERK的表达水平，吉非替尼可以明显抑制照射后的pERK水平。因此，吉非替尼的放射增敏与其抑制pERK的活化能力有关。

#### 1.1.4 抑制放射损伤的再修复

通常放射敏感性主要表现为细胞修复辐射所致DNA损伤的能力，尤其指DNA双链断裂（DSBs）的损伤修复^[5]^。Tanaka等^[5]^采用宿主细胞复活（host cell reactivation, HCR assay）分析技术，将γ线照射后的携带β半乳糖苷酶基因的腺病毒（Ad-βgal）转染至肿瘤细胞内（A549和H1299细胞系），检测Ad-βgal再激活水平从而间接反应肿瘤细胞DNA的损伤修复。经吉非替尼预处理的细胞内Ad-βgal的再激活水平大大下降（*P*=0.006和*P*=0.04）。进而证实吉非替尼是通过抑制细胞对DNA损伤修复能力而产生放射敏感作用。细胞核内γ-H2AX和pNBS1为DSBs产物，亦为DSBs修复起到连接作用^[8]^。免疫荧光显微镜显示，吉非替尼可以延长放射线诱导的细胞核内γ-H2AX和pNBS1焦点的持续时间，从而证实吉非替尼可以抑制DNA双链断裂的修复，并且主要抑制DSBs修复的早期阶段。通过PFGE和中性单细胞凝胶电泳检测发现，吉非替尼并未改变早期DSBs水平，但DNA双链断裂的连接在照射后的前2 h被延迟了。Rad51修复蛋白在DSBs同源重组性修复中起核心作用^[9, 10]^。抑制Rad51表达可以提高放射敏感性^[11]^。Chinnaiyan等^[2]^将H226等细胞系暴露于辐射中，随着暴露时间增加（10 h-24 h）Rad51表达增加。但经厄洛替尼预处理的细胞系中Rad51的表达被明显抑制。

#### 1.1.5 抑制细胞加速再增殖

分次放疗可以促使肿瘤细胞加速再增殖，从而产生放射抗拒。研究表明，这种加速再增殖与EGFR信号旁路的激活有关。在体内大肠癌实验模型中，吉非替尼或放射线单独作用仅可以部分或短暂的抑制肿瘤生长；而吉非替尼联合单次或分次放射治疗后，可以明显抑制肿瘤生长（*P*≤0.001），效果等同于将放射线剂量增加60%^[12]^。在NSCLC异体肿瘤移植模型中，厄洛替尼（0.8 mg/d）联合放疗（12 Gy/2 Gy/21 d, 2 f/w）抑制肿瘤生长的作用可以长达55天^[2]^。因此，EGFR-TKI可以抑制辐射所致的细胞加速再增殖。

#### 1.1.6 抗肿瘤血管生成

目前认为，肿瘤血管的生成在肿瘤的生长和转移方面起到重要作用，并且由于肿瘤血管结构异常导致肿瘤内部乏氧及阻碍药物的进入从而产生治疗抗性。Chinnaiyan等^[2]^分析，厄洛替尼可能通过影响宿主微环境（抑制肿瘤血管生成），改善乏氧从而进一步提高体内放射敏感性。体内外的临床前研究均显示吉非替尼可以抑制血管再生，抑制血管源性生长因子的表达，如VEGF和TGF-β，并降低肿瘤异种移植物的血管数量^[13, 14]^。另外，CDNA微阵列分析显示，厄洛替尼可能通过*Egr-1*、*CXCL1*和*IL-1β*等基因影响放射敏感性^[2]^。

### 1.2 NSCLC细胞系的*EGFR*突变状态对放射敏感性的研究

2006年，Das等^[15]^首次对EGFR突变状态与放射敏感性关系进行了体外研究。该研究选取了19种NSCLC细胞系，其中*EGFR*野生型10种，*EGFR*突变型9种（6种为外显子19突变，3种为外显子21 L858R缺失）。结果显示经射线照射后*EGFR*突变型细胞系，包括对吉非替尼继发耐药的T790M在内，均对放射线敏感；而野生型抗拒。作者分析其原因可能为：辐射诱导的DNA双链断裂的修复延迟，以及凋亡增加。

## 2 临床研究

### 2.1 同步放化疗期间联合EGFR-TKI

CALGB 30106是2002年Ready等^[16]^率先开展的一项吉非替尼联合同步放化疗的临床Ⅱ期研究，并进行了*EGFR*及*K-RAS*基因突变检测。入组的Ⅲ期不可切除NSCLC患者先接受2个周期紫杉醇+卡铂联合每天吉非替尼250 mg口服。按PS评分及体重下降情况分为2组：预后差组（PS=2分或最近3月体重下降≥5%），预后好组（PS=0分-1分或体重下降 < 5%）。第1组接受胸部放疗66 Gy/33 f同期口服吉非替尼，第2组在此基础上联合每周紫杉醇、卡铂方案化疗。该研究入组60例患者（第1组21例，第2组39例），45例患者接受基因检测，*EGFR*突变阳性13例，*K-RAS*突变阳性7例。两组患者中位年龄66岁（41岁-86岁），男性占73%，鳞癌43%、腺癌33%，重度吸烟或未戒烟者89%。除了腹泻及皮疹外，3级以上食管炎及肺炎发生率并未高于同步放化疗（26.2%, 14.8%）。预后好组ORR为81%，预后差组为53%（*P*=0.034）。然而，意想不到的是预后好组疾病无进展生存时间（progression free survival, PFS）为9.2个月，中位生存时间（overall survival, OS）仅13个月；预后差组PFS为13.4个月，中位生存时间竟达19个月。根据基因突变的亚组分析显示：预后好组*EGFR*突变患者中位生存时间仅有7.2个月，明显劣于预后差组*EGFR*突变患者28.4个月；T790M及*K-RAS*突变患者疗效及预后也优于前者，因此该研究并未找到一个提示治疗有效的生物标记物。作者认为，多靶点抑制剂吉非替尼可能通过抑制多条信号通路与同步放化疗产生对抗。因此在Ⅲ期NSCLC非选择人群中建议同步放化疗可与吉非替尼序贯进行。对于预后差或*EGFR*突变阴性的患者接受同步放化疗及吉非替尼治疗有一定前景。该结论还需要临床研究进一步证实。Stinchcombe等^[17]^对同步放化疗期间联合吉非替尼在不可切除Ⅲ期NSCLC中的安全性进行了评估。该研究采用紫杉醇、卡铂、伊立替康三药联合方案诱导化疗后，给予高剂量三维适形放疗（74 Gy）同期紫杉醇、卡铂化疗，联合吉非替尼。共24例患者入组，主要毒副反应为3级食管炎（19.5%）和心律失常（9.5%），3级肺毒性仅为4.8%，安全性可耐受。但生存结果却令人失望，OS仅为9个月，与该中心此前同步放化疗所取得的OS 24个月，5年生存率24%相比明显降低。分析可能原因：①胸部放疗剂量过高：2011年ASTRO年会公布RTOG 0617结果显示，胸部放疗74 Gy高剂量组同步放化疗生存劣于60 Gy标准组；②治疗引起T790M突变，从而导致继发耐药。

另外两项Ⅰ期临床研究^[18, 19]^显示吉非替尼联合同步放化疗治疗局部晚期NSCLC是可行的，但无生存优势。其中3级-4级血液学毒性发生率27%，放射性食管炎发生率22%-27%，3级-5级放射性肺炎发生率约20%。

对于厄洛替尼，Choong等^[20]^进行了一项Ⅰ期临床研究旨在分析厄洛替尼联合两种标准同步放化疗方案（胸部放疗66 Gy，化疗方案EP/PC）治疗不可切除Ⅲ期NSCLC的最大耐受剂量。厄洛替尼仅在同步放化疗期间应用。入选34例患者中，腺癌占21%，女性占41%，全部有吸烟史。结果显示150 mg厄洛替尼安全可耐受，但中位生存时间仅11个月，该研究未能进行EGFR突变检测，无法获得选择人群亚组分析。

Komaki等^[21]^进行的一项Ⅱ期临床试验结果鼓舞人心。厄洛替尼（150 mg/d）联合标准同步放化疗（每周紫杉醇+卡铂方案，胸部放疗63 Gy/35 f）应用于不可手术Ⅲ期NSCLC患者，48例患者入组。无4级-5级毒性反应出现，3级毒性反应发生率也很低（痤疮2例，放射性肺炎3例，放射性食管炎1例）。PFS及OS分别为13.6个月和25.8个月。更值得注意的是，该组人群中仅12%存在*EGFR*突变，提示厄洛替尼与放化疗联合可以提高疗效，分析可能与其引起细胞周期阻滞、诱导细胞凋亡、降低治疗期间放射抗拒等机制有关。

2011年瑞士第十四届肺癌大会上，一项研究^[22]^报道了在非指定NSCLC人群中，厄洛替尼联合同步放化疗的Ⅱ期临床研究结果。入组患者化疗采用一线标准新药吉西他滨或培美曲塞，同期口服厄洛替尼，全部患者均接受总剂量为50.4 Gy-59.4 Gy的常规分割胸部放疗。结果有60例患者入组，20例鳞癌、40例腺癌，Ⅱ期-Ⅲ期占63%、单个转移灶的Ⅳ期患者占27%、术后局部复发占10%。吉西他滨组23例，培美曲塞组37例。3级-4级食管炎及肺炎发生率分别为2%、5%。吉西他滨组与培美曲塞组客观缓解率分别为64%、68%。两组中位生存时间14.4个月。亚组分析显示，培美曲塞组非鳞癌患者PFS（7.5个月）、OS（14.4个月）更优。但全身治疗是否与放疗同时进行，该报道并未明确指出。

EGFR和VEGF抑制剂均可以改善NSCLC生存，并且阻断这些通路也显示了放射增敏效果。Socinski等^[23]^报道了同步放化疗联合吉非替尼与贝伐珠单抗的Ⅰ/Ⅱ期临床研究。入组Ⅲ期NSCLC患者先接受贝伐珠单抗及紫杉醇、卡铂诱导化疗，之后进行74 Gy胸部适形放疗同步PC化疗，第1组同期行贝伐珠单抗，第2、3组同时还口服厄洛替尼，剂量分别为100 mg、150 mg。该研究首要终点为1年无疾病进展率，包括鳞癌在内所有病理类型均可入组。截止发表当时，共31例患者入组，12例鳞癌，19例非鳞癌。总疾病缓解率为68.2%，1年无进展生存率为58%，1年总生存率估计为79%，数据可观。诱导治疗期间仅1例出现3度高血压，但同步治疗期间急性食管炎发生率较高，安全性评估及生存有待进一步观察。然而，根据2011年第十四届世界肺癌大会发布的最新数据显示，与过去同方案的同步放化疗相比，加入厄洛替尼和贝伐珠单抗后，无疾病进展生存和总生存结果相似。鳞癌患者因肺出血发生率高而被停止入组。3级及以上食管炎、肺炎发生率分别为29%、2%，3度以上中性粒细胞下降及血小板下降分别为13%、7%。1年无进展生存率44%，中位PFS 10个月，中位OS 19个月。目前该临床研究（NCT00280150）已结束，让我们拭目以待^[24]^。

### 2.2 EGFR-TKI同期联合放疗

日本的Okamoto等^[25]^开展了一项关于评估一线吉非替尼联合胸部放疗治疗局部晚期NSCLC的可行性研究。不可切除的Ⅲ期NSCLC患者吉非替尼250 mg/d口服14 d后，开始胸部三维适形放疗GTV 60 Gy/30 f，V20≤35%。放疗同期及结束后继续使用吉非替尼，直到疾病进展。2003年8月-2005年9月，共2年时间仅9例患者入组，中位年龄63岁（56岁-71岁），Ⅲa期5例，Ⅲb期4例，腺癌6例，男性8例均为重度吸烟患者。然而，2例患者在口服前2周吉非替尼的过程中出现疾病进展，3例在放疗期间出组（2例出现肺毒性，1例PD），因此该临床试验提前关闭，完成治疗的患者仅4例，疗效均为PR，其中2例出现了治疗相关的肺炎（1级/3级）。8例患者进行了*EGFR*基因突变检测，2例*EGFR*突变阳性，并且均完成治疗，评效PR，2例生存期均超过5年。该研究教训多于经验：①单药吉非替尼诱导治疗不宜用于Ⅲ期NSCLC，不足以控制疾病；②日本曾报道吉非替尼所致威胁生命的间质性肺炎（interstitial lung disease, ILD）高危因素为男性、吸烟史及先天性肺纤维化^[26]^。该研究治疗相关的肺炎发生率较高（57%），作者推测与入组患者男性、吸烟比例较高有关。③该研究中2例*EGFR*突变患者均完成治疗并取得了极好的临床效果，提示*EGFR*突变的局部晚期NSCLC患者吉非替尼联合胸部放疗是铂类为基础的同步放化疗的潜在可替代方案。

JCOG 0402^[27]^研究亦带来令人振奋的生存结果，但其较高的肺毒性让人喜忧参半。该研究为一项Ⅱ期临床试验，入组患者38例，诺维本+顺铂诱导化疗后给予吉非替尼联合胸部放疗60 Gy/30 f。OS为28.1个月。但仅有61%患者未并发≥2级的肺炎，14例患者因一过性转氨酶升高（3级-4级）延迟靶向治疗。

我国夏廷毅等^[28]^开展了一项EGFR-TKI同步个体化放疗治疗晚期/转移性NSCLC的Ⅱ期临床研究。胸部放疗采用3DCRT、IMRT（常规分割）及γ-SBRT（大分割）。GTV边缘中位剂量70 Gy，中位生物等效剂量105 Gy，放疗计划V20≤30%。同期口服吉非替尼或厄洛替尼。入组患者26例，其中21例为Ⅳ期、5例为Ⅲ期，腺癌19例、鳞癌4例，男性11例、女性15例，不吸烟患者9例。12例为初治患者。4度中性粒细胞下降1例（4%），血小板下降2例（8%），3级以上放射性肺炎和食管炎发生率均为4%，耐受性良好。胸部肿瘤局部控制率96%，中位PFS为10.2个月，中位TTP为6.3个月，中位生存期为21.8个月。1年、2年、3年OS分别为57%、45%、30%。因此该种联合治疗模式安全有效。台北Chang等^[29]^对EGFR-TKI同步断层放疗治疗Ⅲb（2例）期/Ⅳ（23例）期非鳞NSCLC进行了回顾性分析。一线厄洛替尼或吉非替尼有效情况下，后续联合使用断层放疗（针对多个转移灶进行放疗，40 Gy-50 Gy/16 f-20 f）。最常见的不良反应是Ⅰ度-Ⅱ度乏力，3级以上SRP发生率12%（3/25），其中2例死亡。TKI联合断层放疗后总有效率为84%。中位PFS 16个月，1年、2年、3年生存率分别为92%、82.5%、62.5%。亚组分析显示，PFS > 10个月的患者OS有优势（*P* < 0.000, 1）。根据以上两个我国开展的临床研究结果，EGFR-TKI联合胸部及转移灶放疗治疗转移性NSCLC极具前景，对于局部晚期NSCLC此种联合模式亦可作为无法耐受放化疗时的替代方案。

### 2.3 EGFR-TKI维持治疗

意义重大的SWOG 0023^[30]^基本回答了吉非替尼维持的意义。西南肿瘤协作组（Southwest Oncology Group, SWOG）在基于SWOG 9504的Ⅱ期临床研究——同步放化疗后多西他赛巩固治疗可以改善患者生存，设计了SWOG 0023——Ⅲ期NSCLC接受同步放化疗（EP, 61 Gy）+多西他赛巩固治疗后无进展者进行吉非替尼或安慰剂维持治疗Ⅲ期临床研究。包含573例患者的分析显示，吉非替尼组（*n*=118）较安慰剂组（*n*=125）中位生存期短（23个月*vs*. 35个月，*P*=0.013）。因此，对于Ⅲ期NSCCLC非选择人群吉非替尼的维持治疗并未带来生存获益，由此使得同期及之后的临床试验停止了对患者进行吉非替尼维持治疗。

厄洛替尼的维持治疗方面，Rigas等^[31]^开展了一项随机对照临床研究对Ⅲ期不可切除NSCLC同步放化疗后厄洛替尼2年维持治疗和口服安慰剂进行了比较。胸部采用常规放疗，总剂量为61 Gy，同期化疗方案为多西他赛联合卡铂，治疗结束3周-7周后开始口服厄洛替尼或安慰剂150 mg/d，共2年。结果243例患者入组，其中女性占41%，腺癌31%、鳞癌44%，Ⅲb期44%，PS为0分-1分96%，15%患者体重下降≥10%。厄洛替尼维持治疗组无明显毒性反应，耐受性良好。主要研究终点PFS，厄洛替尼组与安慰剂组分别为13.5个月*vs* 10.4个月（*P*=0.12）；次要研究终点OS，两组分别为30.4个月*vs* 25.1个月（*P*=0.20）；另一研究终点为TTP，截止发表厄洛替尼组中位TTP尚未达到，而安慰剂组为13.1个月（*P*=0.005）。该研究提示不可切除的Ⅲ期NSCLC同步放化疗后应用厄洛替尼维持可以延长TTP，笔者考虑可能与厄洛替尼抑制放射损伤的再修复有关。但PFS与OS无明显延长。

另外，北京大学肿瘤医院朱广迎等^[32, 33]^回顾性分析了Ⅲ期NSCLC中*EGFR*突变状态对放化疗疗效和生存的预示，其中鳞癌患者中意义更大。

## 3 结语

综上所述，EGFR-TKI通过引起细胞周期阻滞、诱导细胞凋亡、降低放射抗拒、抑制放射损伤的再修复、抑制细胞加速再增殖等机制增强了NSCLC细胞系的放射敏感性。然而临床应用中，吉非替尼与厄洛替尼的增敏效果需区别对待，而靶向药物与放疗的结合模式亦需探索。对于局部晚期NSCLC同步放化疗期间联合EGFR-TKI结果并不令人满意，但改变化疗方案是否可以取得更好的疗效尚需研究进一步证实。对于晚期NSCLC，EGFR-TKI联合转移灶放疗极具前景，但联合胸部放疗所致间质性肺炎的发生仍是不可忽视的问题。同步放化疗后的维持治疗中：吉非替尼没有价值，而厄洛替尼与安慰剂疗效相当、耐受性尚可。目前尚有数个Ⅰ期/Ⅱ期临床试验正在进行（NCT01391260、NCT00563784、NCT00888511等）^[34]^。我们相信随着基础与临床研究的不断深入，这些谜团将逐步解开，EGFR-TKI靶向治疗与放疗联合在晚期NSCLC中将展现出更为广阔的前景。
